# Does Osteopathic Heart-Focused Palpation Modify Heart Rate Variability in Stressed Participants with Musculoskeletal Pain? A Randomised Controlled Pilot Study

**DOI:** 10.3390/healthcare12020138

**Published:** 2024-01-08

**Authors:** Torsten Liem, Lucas Bohlen, Anna-Moyra Jung, Samira Hitsch, Tobias Schmidt

**Affiliations:** 1 Osteopathic Research Institute, 22297 Hamburg, Germany; 2 Research Department, Osteopathie Schule Deutschland, 22297 Hamburg, Germany; 3 Department of Healthcare, Dresden International University, 01067 Dresden, Germany; 4 Institute of Interdisciplinary Exercise Science and Sports Medicine, MSH Medical School Hamburg, 20457 Hamburg, Germany

**Keywords:** osteopathic manipulative treatment, mind–body therapies, psychosomatic osteopathy, heart-focused palpation, stress, heart rate variability, autonomic nervous system, musculoskeletal pain, complementary medicine, alternative medicine

## Abstract

Heart rate variability (HRV) describes fluctuations in time intervals between heartbeats and reflects autonomic activity. HRV is reduced in stressed patients with musculoskeletal pain and improved after osteopathic manipulative treatment and mind–body interventions. Heart-focused palpation (HFP) combines manual and mind–body approaches to facilitate relaxation. This randomised controlled pilot study investigated the feasibility and sample size for a future randomised controlled trial and the effect of a single treatment with HFP or sham HFP (SHAM) on short-term HRV. A total of Thirty-three adults (47.7 ± 13.5 years old) with stress and musculoskeletal pain completed the trial with acceptable rates of recruitment (8.25 subjects per site/month), retention (100%), adherence (100%), and adverse events (0%). HFP (*n* = 18), but not SHAM (*n* = 15), significantly increased the root mean square of successive RR interval differences (*p* = 0.036), standard deviation of the NN intervals (*p* = 0.009), and ratio of the low-frequency to high-frequency power band (*p* = 0.026). HFP and SHAM significantly decreased the heart rate (*p* < 0.001, *p* = 0.009) but not the stress index and ratio of the Poincaré plot standard deviation along and perpendicular to the line of identity (*p* > 0.05). A power analysis calculated 72 participants. Taken together, the study was feasible and HFP improved HRV in stressed subjects with musculoskeletal pain, suggesting a parasympathetic effect.

## 1. Introduction

Musculoskeletal pain (MP), like low back pain (LBP), is prevalent and disabling [1,2] and seems to be associated with elevated stress levels [3,4]. Stress (defined as “the nonspecific response of the body to any demand“ [5]) activates the hypothalamus–pituitary–adrenal (HPA) axis and the sympathetic–adreno–medullar (SAM) axis [6], leading to an increase in cortisol levels (HPA axis activity) and a decrease in heart rate variability (HRV) (SAM axis activity) [7,8]. Notably, cortisol is a steroid hormone that is secreted during stress and reflects the stress response [9], whereas HRV refers to the variation in time intervals between heartbeats and reflects autonomic activity [10]. Consequently, patients with MP like LBP seem to show aberrant cortisol levels [11] and reduced HRV [12] compared to healthy controls. Although simplified, reduced HRV seems to reflect increased and decreased sympathetic and parasympathetic nervous system (SNS and PNS) activity, respectively [10,13]. Importantly, HRV comprises various parameters and those relevant to this study are defined and interpreted in Table 1. Furthermore, evidence suggests that HRV (e.g., root mean square of successive RR interval differences (RMSSD)) is associated with stress [8,14] and arguably cortisol levels [15]. Hence, patients with MP like LBP demonstrate elevated cortisol levels and reduced HRV, suggesting increased stress levels as well as increased SNS and/or decreased PNS activity [4,11,12].

The management of MP like LBP involves treatment approaches ranging from pharmacological and surgical to behavioural and non-pharmacological interventions [16,17]. Still, non-pharmacological interventions are generally preferred [18,19], which arguably include osteopathic manipulative treatment (OMT) and mind–body interventions (MBI). On the one hand, OMT can be defined as a person-centred approach to healthcare that applies manual diagnosis and treatment and provides psychosocial support and advice on nutrition, exercise, and lifestyle [20]. On the other hand, MBI aims to improve the interaction of body and mind (e.g., through changes in breathing rhythm, body movements, and mental status) [21] and comprises diverse approaches including yoga, meditation, mindfulness, and tai chi [22]. The current body of evidence suggests that both OMT and MBI improve MP [23,24] like LBP [25,26]. However, although OMT and MBI seem to improve MP, the underlying mechanisms are not fully understood [25,27]. To date, various putative mechanisms are discussed, which involve changes in autonomic activity [28,29]. In sum, OMT and MBI were shown to improve MP [23,24], which may involve autonomic mechanisms [28,29].

Indeed, both OMT and MBI may improve stress and cortisol levels as well as autonomic activity and HRV. On the one hand, OMT was shown to improve stress and cortisol levels in some studies [30,31] but not others [32,33,34]. A recent systematic review concluded that OMT decreases cortisol levels with a medium effect [35]. Similarly, reviews have shown that OMT improves autonomic activity [36,37] and HRV [38,39]. More specifically, most studies report a significant effect of OMT on HRV by means of decreasing the low-frequency power band (LF) and ratio of the low-frequency to high-frequency power band (LF/HF) and increasing the high-frequency power band (HF), standard deviation of the NN intervals (SDNN), and RMSSD [28,40,41,42,43,44], which indicates an increase in parasympathetic and a decrease in sympathetic activity [45,46]. However, counterevidence is available showing no significant effect of OMT on HRV [31,47,48,49,50]. Interestingly, OMT seems to alter ANS activity in patients with stress [51]. On the other hand, MBIs were shown to reduce stress and cortisol levels in most reviews (after meditation, mindfulness, yoga, and/or tai chi) [52,53,54], whereas some have reported inconsistent results for changes in cortisol levels (after mindfulness) [55]. Similarly, MBIs were shown to improve HRV by decreasing LF and LF/HF and increasing HF, SDNN, and RMSSD in some reviews (after mindfulness, yoga, and/or tai chi) [54,56], while others showed no changes in LF, HF, LF/HF, RMSSD, and SDNN (after mindfulness) [57]. Nonetheless, the literature on the effect of OMT and MBI on stress and cortisol levels as well as autonomic activity and HRV remains partially limited and heterogenous, and future research is needed [35,36,54,58]. Taken together, OMT and MBI were shown to reduce stress (by means of decreased cortisol levels) and enhance autonomic activity (by means of increased HRV) [35,37,54,58].

Despite the altered stress levels and autonomic activity in MP and the efficacy of OMT and MBI to improve MP as well as stress levels and autonomic activity (cortisol levels and HRV), few interventions and studies have combined approaches from both fields for the treatment of patients with MP reporting stress. Nonetheless, the integration of manual and mind–body interventions has been called for [59] and a few studies have already investigated the feasibility of combining manual and psychological approaches for chronic pain patients [60,61]. In this study, a novel osteopathic technique was applied, namely, heart-focused palpation (HFP). HFP emerged as part of a new osteopathic approach termed psychosomatic osteopathy (PSO), which was developed in clinical practice between 2016 and 2018 and published subsequently [62,63,64]. HFP combines OMT approaches (i.e., touch and manipulation) with modalities from MBI (i.e., changes in breathing and mental focus) [62]. The aim of HFP is to facilitate relaxation of the body and mind by reducing stress and improving autonomic activity [62]. The approach may engage both top-down and bottom-up mechanisms (between peripheral tissues and the nervous system) [62], arguably via autonomic pathways (suggesting HRV as a marker) [65]. Before this background, we hypothesise that HFP (integrating OMT and MBI modalities) may improve HRV (characterising autonomic activity) in stressed subjects with MP.

Herein, we aimed to investigate the effect of HFP on the HRV of stressed subjects with MP. The primary objective was to assess the feasibility of the study protocol for a future randomised controlled trial (RCT) by evaluating the recruitment, retention, adherence, and safety of the trial. To be considered feasible, at least 30 subjects must be recruited at a rate of more than 7.5 subjects per site per month, the retention and adherence rates must exceed 80%, and the adverse events rate must be less than 6%. The secondary objective was to evaluate the effect of HFP compared to sham treatment imitating HFP (SHAM) on the HRV of stressed participants with MP by means of the heart rate (HR), RMSSD, SDNN, stress index (SI), LF/HF, and the ratio of the Poincaré plot standard deviation along and perpendicular to the line of identity (SD2/SD1). It was hypothesised that HFP leads to a decrease in HR, SI, LF/HF, and SD2/SD1 as well as an increase in SDNN and RMSSD. These predicted changes reflect increased parasympathetic activity [10,36] and were previously reported in patients with chronic LBP (CLBP) after OMT [42]. The tertiary objective was to conduct a power analysis to calculate the sample size that will be required for a future RCT.

## 2. Materials and Methods

### 2.1. Study Design

A single-blinded, multicentre, parallel-group, randomised controlled pilot study was conducted and reported according to the CONSORT statement for pilot and feasibility trials (Table S1) [66]. The study protocol was prospectively approved by the ethics committee of the Osteopathic Research Institute (Nr.: 019-12) and retrospectively registered in the German Clinical Trials Register (DRKS00023730).

### 2.2. Participants

Individuals with MP and self-perceived stress were recruited from two private osteopathy practices in Switzerland and Germany. Participants were recruited from two countries to increase the generalisability of the results [67]. During acquisition, information about the study was provided on the websites of both practices, shared through notices and flyers, advertised in local newspapers, and passed on through word of mouth. According to the inclusion criteria, participants needed to be (1) adults (>18 years and <70 years old); (2) able to provide informed consent (presupposing sufficient language skills in German); (3) symptomatic (reporting MP, independent of the location and duration); and (4) stressed (self-perceived stress level of ≥12 on the German version of the 10-item Perceived Stress Scale (PSS-10)) [68,69] (Appendix A). The exclusion criteria defined participants as being ineligible if they reported (1) cardiac arrhythmias (e.g., extrasystole or atrial fibrillation); (2) implanted pacemakers; (3) diseases of the heart, blood vessels, or lungs; (4) neurological, psychiatric, or other serious disorders; (5) intake of mediations (particularly those affecting the cardiovascular or nervous system); and (6) pregnancy [70].

### 2.3. Interventions

Participants were randomly allocated into an intervention and control group, which received a single treatment session of either HFP (HFP group) or sham treatment imitating HFP (SHAM group). The interventions were carried out by two female osteopaths with approximately one year of practice experience and five years of education (under- and postgraduate). The therapists (AMJ and SH) were trained to apply HFP during a three-day course (provided by TL), which comprised consensus training (to ensure that the therapists perform the techniques coherently).

#### 2.3.1. HFP

**Figure 1:**
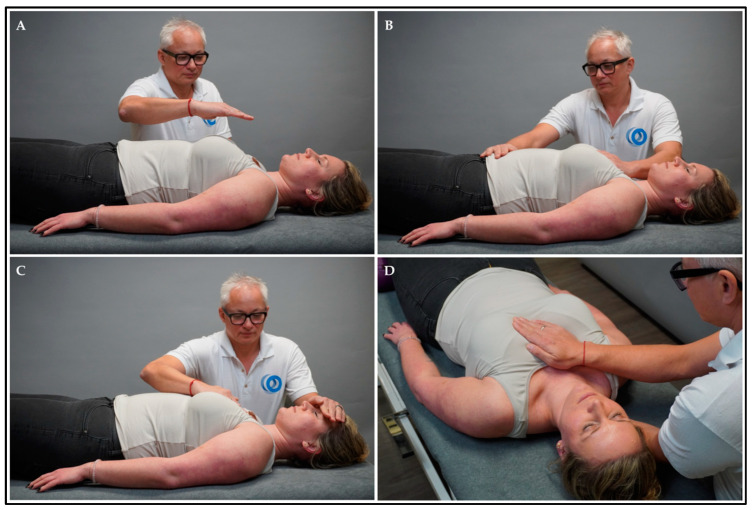
HFP intervention. Explanation: The therapist placed (**A**) one hand above the anterior chest area and slowly moved it to the body surface (i.e., sternum); (**B**) one hand on the sternum and the other hand on the abdomen (i.e., below the umbilicus); (**C**) one hand on the sternum and the other hand on the head (i.e., forehead); and (**D**) one hand on the sternum and the other hand on the posterior chest area (i.e., mid-thoracic spine).

The intervention group received treatment by means of HFP for 15–20 min (HFP group). HFP combines manual approaches with modalities from MBIs. The participant lay in a supine position on the treatment bench and the therapist sat or stood beside the treatment bench. First, the participant was asked to actively perceive (but not rate) current body sensations regarding arousal, vitality, and feelings (~2 min). Second, the therapist placed one hand in the air above the anterior chest area and slowly moved it to the body surface (~2 min) (Figure 1A). Third, the therapist placed one hand on the chest (on the sternum) and laid the other hand on the abdomen (below the umbilicus) and head (on the forehead), respectively (~2 min) (Figure 1B,C). While the therapist palpated these regions (following micro-movements), the participant was instructed to attend to pleasant sensations from these body regions and shift the attention between regions in unison with the breathing cycles. Fourth, the therapists palpated the heart region (i.e., the area of the thorax where the heart is situated) (Appendix B). Subsequently, one hand was placed on the sternum (anterior thorax) and the other hand was placed on the mid-thoracic spine (posterior thorax) (~9–14 min) (Figure 1D). During palpation, the therapist attended to the heartbeat, perceived the heat emission, visualised the heart in three dimensions, and palpated tensions in the tissue. Using manual pressure, the tissue between the hands was passively tested and actively moved using the following parameters: anterior, posterior, cranial, caudal, and lateral motion, rotation, or inclination. The mental status of both the therapist and participant was focused on perceiving the heart area. The treatment was terminated if tension in the tissue was reduced, and the micro-movements reached temporary stillness. During the treatment, eye contact was repeatedly established between subjects and the therapist, and the participants were instructed to alter their breathing rate (slow down, deepen, and attend to the breath) and to actively perceive and accept bodily sensations non-judgementally [62].

#### 2.3.2. SHAM

**Figure 2:**
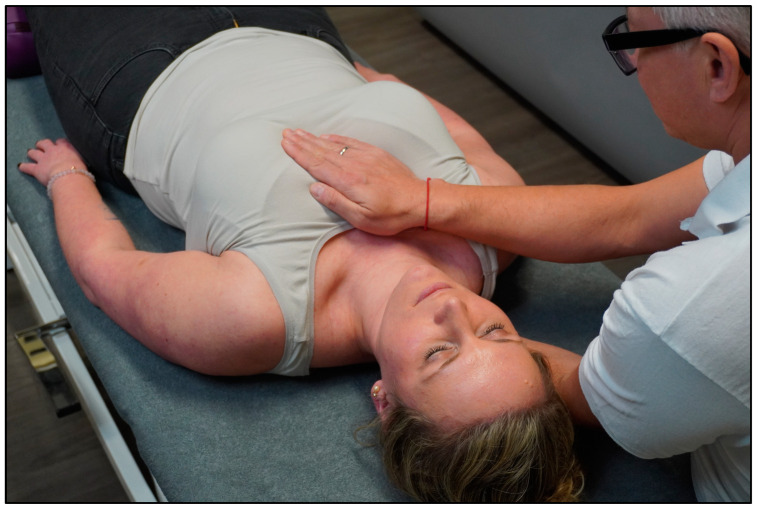
SHAM intervention.

The control group received sham treatment that imitates HFP for 15–20 min (SHAM group). The participant lay in a supine position on the treatment bench and the therapist sat on a chair at the head end of the treatment bench. The therapist placed one hand on the sternum (anterior thorax) and the other hand was placed on the mid-thoracic spine (posterior thorax) (~15–20 min) (Figure 2). During the manual contact, the therapist did not aim to treat the participant in the SHAM group. More specifically, there was no (1) intention to treat; (2) focus on tactile sensations; (3) verbal communication; and (4) application of osteopathic tests or techniques. Notably, the sham intervention did not comprise modalities from MBIs (e.g., no changes in breathing rate, cognitive status, etc.).

### 2.4. Outcomes

#### 2.4.1. Feasibility

Primarily, the feasibility of the study protocol was evaluated. Therefore, the rates of recruitment, retention, adherence, and adverse events (AEs) were calculated. The recruitment of the trial was considered feasible if at least 30 subjects could be recruited at two sites over the course of two months, which requires recruiting at least 7.5 subjects per site per month (recruitment rate: >7.5). Furthermore, the study retention and adherence were considered feasible if at least 80% of the recruited participants completed the trial (retention rate: >80%) and adhered to the treatment sessions (adherence rate: >80%) [71]. Lastly, the safety of the study was assessed by asking participants to report harms that occurred during and up to one month after the trial to the investigators by phone (including the type, severity, frequency, duration, and attributed cause of AEs as well as actions taken against AEs). Based on previous research [72], the study was considered safe if less than 6% of participants report mild or moderate AEs (adverse events rate: <6%). The study must be stopped if serious AEs occur. More specifically, the feasibility rates were calculated as follows: (1) recruitment rate: the number of subjects recruited at baseline was divided by the number of recruitment sites and divided by the number of months of recruitment [73]; (2) retention rate: the number of subjects analysed at the endpoint was divided by the number of subjects recruited at baseline and multiplied by 100 [74]; (3) adherence rate: the number of treatment sessions attended was divided by the number of treatment sessions available and multiplied by 100 [75]; and (4) adverse events rate: the number of AEs was divided by the number of subjects analysed at the endpoint and multiplied by 100 [76]. Beyond the recruitment, retention, adherence, and adverse events rates, the investigators subjectively considered guiding questions for feasibility studies [77]. Lastly, the overall feasibility of the study protocol was rated as (1) not feasible; (2) feasible with modifications; (3) feasible with monitoring; or (4) feasible as is [78].

#### 2.4.2. HRV

Secondarily, the effect of HFP on the HRV of stressed participants with MP was measured using the “HRV Scanner Standard” from BioSign [70]. HRV is a non-invasive and pain-free measurement method that is affordable, quick, and easy to use [79]. It is a valid and reliable method to evaluate the effect of therapeutic interventions on the activity of the ANS [80]. More specifically, HRV can be used to assess the effect of OMT on the ANS [39]. In this study, a short-term (five-minute) measurement was conducted (pre- and post-intervention), and the following parameters were assessed: (1) heart rate (HR); (2) root mean square of successive RR interval differences (RMSSD); (3) standard deviation of the NN intervals (SDNN); (4) stress index (SI); (5) ratio of the low-frequency (LF) to high-frequency (HF) power band (LF/HF); and (6) ratio of the Poincaré plot standard deviation along (SD2) and perpendicular (SD1) to the line of identity (SD2/SD1) [70] (Table 1).

**Table 1:** Heart rate variability parameters [10,36,70].

Parameter	Definition	Autonomic Activity
HR (bpm)	Number of heart beats per minute	HR ↑ = SNS ↑
RMSSD (ms)	Root mean square of successive RR interval differences	RMSSD ↑ = PNS ↑
SDNN (ms)	Standard deviation of the NN intervals	SDNN ↑ = PNS ↑
SI (pts)	Stress index according to Baevsky	SI ↑ = SNS ↑
LF/HF (ratio)	Ratio of the low-frequency (LF) to high-frequency (HF) power band	LF/HF ↑ = SNS ↑
SD2/SD1 (ratio)	Ratio of the Poincaré plot standard deviation along (SD2) and perpendicular (SD1) to the line of identity	SD2/SD1 ↑ = SNS ↑

Abbreviations: HR = heart rate; RMSSD = root mean square of successive RR interval differences; SDNN = standard deviation of the NN intervals; SI = stress index; LF = low-frequency power band; HF = high-frequency power band; LF/HF = ratio of the low-frequency to high-frequency power band; SD2/SD1 = ratio of the Poincaré plot standard deviation along and perpendicular to the line of identity; SNS = sympathetic nervous system activity; PNS = parasympathetic nervous system activity; ↑ = increase; bpm = beats per minute; ms = milliseconds; pts = points. Explanation: The definition of RMSSD and SDNN include the terms RR and NN intervals, respectively. Both RR and NN refer to the inter-beat interval between two heartbeats, more specifically, the peaks of the R waves in the electrocardiogram. However, while RR considers all R peaks, NN only considers normal R peaks (without artifacts) [10].

#### 2.4.3. Power Analysis

Tertiarily, the sample size for a future RCT was calculated with an Excel tool from ACOMED statistics [81,82].

### 2.5. Sample Size

The sample size was planned with 30 participants according to the rule of thumb for pilot studies [83].

### 2.6. Randomisation

Participants were randomly allocated into the HFP or SHAM group by drawing lots (1:1 ratio). This simple randomisation was carried out separately for each location (Switzerland and Germany) by different investigators (AMJ and SH) at the same time.

### 2.7. Blinding

Participants and statisticians were blinded to the conditions, but not the investigators (being therapists and assessors). Notably, therapists cannot be blinded in manual therapy trials because they are necessarily aware of the technique they apply with their hands (double-blinding is not achievable) [84].

### 2.8. Procedure

The study was conducted in two private practices for osteopathy in Switzerland and Germany. All sessions were carried out between 8 a.m. and 6 p.m. The room temperature was approximately 22 °C. At baseline, participants signed the informed consent, provided personal and demographic information, and filled in the stress scale (PSS-10). Afterward, the participants underwent one treatment session with two measurements (pre- and post-intervention). Then, subjects lay down on a treatment bench (supine position) and were instructed to stay relaxed and breathe normally. After five minutes of relaxation (to achieve an autonomic resting state), participants were connected to the HRV device (electrodes were attached to the wrists and earlobes). Subsequently, participants (1) were measured for five minutes (pre-intervention), (2) received treatment using HFP or SHAM for 15–20 min (intervention), and (3) were measured again for five minutes (post-intervention). Thereafter, the electrodes and ear clips were detached, and the session was finished.

### 2.9. Statistics

The primary (feasibility) outcomes were calculated as numbers (*n*) and percentages (%). The secondary (HRV) outcomes were assessed descriptively using mean (*M*) and standard deviation (*SD*). Normal distribution of the data was assessed using the Kolmogorov–Smirnov test. If the data were distributed normally, the mean values were compared (1) between groups before treatment (pre-intervention) using the independent two-sample Student’s *t*-test (between-participants); and (2) within each group from before to after treatment (pre- to post-intervention) using the paired Student’s *t*-test (within-participants). If the data were not distributed normally, significant differences were assessed using the Wilcoxon signed-rank test for related samples. For categorical data, a Chi-square test was conducted. The *p*-values were calculated with two-sided tests and the significance level was set to *p* < 0.05 (significant) and *p* < 0.01 (highly significant). Effect sizes were calculated using Cohen’s *d* and were defined as small (0.2), medium (0.5), and large effects (0.8) [85]. Calculations for the primary and secondary outcomes were carried out with IBM SPSS Statistics (Version 22). The tertiary outcome was calculated with an Excel tool [81] based on Lachin (1981) [82].

## 3. Results

### 3.1. Participant Flow

**Figure 3:**
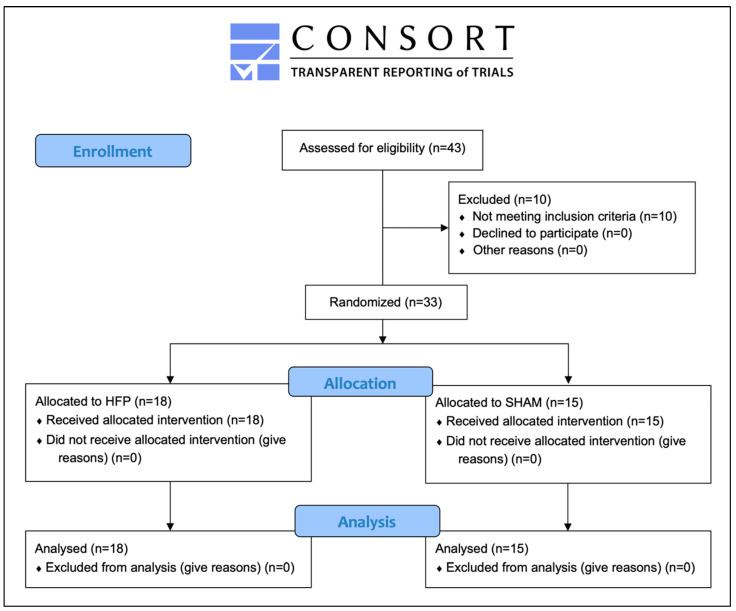
Participant flow diagram.

Overall, 43 people were assessed for eligibility and 10 individuals were excluded from participation. Of those, five people did not report MP and five people did not demonstrate the required stress level (PSS-10: <12). Hence, 33 participants were recruited and randomised for this study (76% of the subjects that were assessed for eligibility). Over the course of the trial, no participants dropped out and data from 33 participants were analysed (Figure 3).

### 3.2. Recruitment

Participants were recruited between November and December 2019. The study ended after the treatment session.

### 3.3. Baseline Data

Normality was tested with the Kolmogorov–Smirnov test and the data were not normally distributed for sex (D[34] = 0.383, *p* < 0.001). Most demographic and clinical data demonstrated no significant difference between HFP and SHAM groups at baseline (*p* > 0.05) (Table 2). However, the SHAM group demonstrated significantly higher RMSSD (*p* = 0.007) and SDNN (*p* = 0.036) compared to the HFP group, which limits the inter-group comparability. Participants reported musculoskeletal pain in the cervical spine (HFP: 4, SHAM: 2), thoracic spine (HFP: 0, SHAM: 1), lumbar spine (HFP: 5, SHAM: 6), upper extremity (HFP: 4, SHAM: 4), lower extremity (HFP: 3, SHAM: 1), or multiple locations (HFP: 2, SHAM: 1).

### 3.4. Feasibility

In this study, 33 subjects were recruited at two sites over two months (recruitment rate: 8.25), showing no dropouts from the trial (retention rate: 100%), no non-compliance with the treatment sessions (adherence rate: 100%), and no harms (adverse events rate: 0%) (Table 3). Beyond the recruitment, retention, adherence, and adverse events rates, some guiding questions by Orsmond et Cohn (2015) [77] were considered to determine feasibility. Accordingly, the pilot study demonstrated suitable outcome measures, reasonable time burden, feasible trial management, acceptable data handling, and ethical and effective interventions. Taken together, the study protocol appears feasible, pending some modifications [78], which are discussed subsequently.

### 3.5. Heart Rate Variability

Normality was tested with the Kolmogorov–Smirnov test. The data were not normally distributed for RMSSD at pre-intervention (D[34] = 0.183, *p* = 0.05) and LF/HF at pre-intervention (D[34] = 0.256, *p* < 0.001) and post-intervention (D[34] = 0.265, *p* < 0.001). Thus, RMSSD and LF/HF were assessed with the Wilcoxon signed-rank test. All data for the secondary outcomes were analysed and significant effects of HFP and SHAM on HRV were detected (Table 4). In detail, there was a significant within-group difference from pre- to post-intervention, showing a decrease in (1) HR after both HFP (*M* = −2.8; *SD* = 2.2; *p* < 0.001; Cohen’s *d* = 0.303) and SHAM (*M* = −2.5; *SD* = 3.2; *p* = 0.009; Cohen’s *d* = 0.191). Moreover, the within-group difference from pre- to post-intervention showed a significant increase in (2) RMSSD after HFP (*M* = 23.8; *SD* = 44.4; *p* = 0.036; Cohen’s *d* = −0.519) but not SHAM (*M* = 3.5; *SD* = 20.6; *p* = 0.521; Cohen’s *d* = −0.094); and (3) SDNN after HFP (*M* = 17.8; *SD* = 25.4; *p* = 0.009; Cohen’s *d* = −0.772) but not SHAM (*M* = 6.3; *SD* = 18.2; *p* = 0.200; Cohen’s *d* = −0.229). In contrast, no significant within-group differences were reported from pre- to post-intervention for (4) SI after HFP (*M* = −44.4; *SD* = 104.4; *p* = 0.089; Cohen’s *d* = 0.384) and SHAM (*M* = −35.4; *SD* = 69.6; *p* = 0.069; Cohen’s *d* = 0.292). However, there was a significant within-group difference from pre- to post-intervention, showing an increase in (5) LF/HF after HFP (*M* = 3.3; *SD* = 5.7; *p* = 0.026; Cohen’s *d* = −0.635) but not SHAM (*M* = 0.4; *SD* = 1.6; *p* = 0.359; Cohen’s *d* = −0.306). In turn, no significant within-group differences were reported from pre- to post-intervention for (6) SD2/SD1 after HFP (*M* = −0.1; *SD* = 1.0; *p* = 0.527; Cohen’s *d* = 0.103) and SHAM (*M* = 0.0; *SD* = 0.74; *p* = 0.875; Cohen’s *d* = −0.160).

### 3.6. Power Analysis

The required sample size for a future RCT was calculated with an Excel tool [81] based on Lachin (1981) [82]. The power analysis was carried out using the HRV parameter RMSSD. Based on an assumed type I error level of 0.05 (one-sided), a statistical power of 80%, and a mean difference of 23.8 for HFP and 3.5 for SHAM, a total sample size of 66 participants was calculated. Considering an estimated drop-out of 10%, the sample size for a future RCT was determined to be 72 participants.

## 4. Discussion

### 4.1. Overview of Findings

This pilot study aimed to (1) evaluate the feasibility of the study protocol, (2) investigate the effect of HFP on HRV in stressed participants with MP, and (3) calculate the sample size required for a future RCT. Overall, 43 participants were screened for eligibility and 33 were randomised and analysed. The sample showed no significant between-group differences in demographic data (age, sex, height, and weight) and clinical data (PSS-10 score and HRV parameters: HR, SI, LF/HF, and SD2/SD2) at baseline (*p* > 0.05), except for significantly higher RMSSD (*p* = 0.007) and SDNN (*p* = 0.036) in the SHAM, compared to the HFP, group. Therefore, an acceptable intergroup comparability can be assumed for all measures but not for RMSSD and SDNN.

The primary objective was to assess the feasibility of the study protocol for a future RCT by evaluating recruitment, retention, adherence, and safety. The recruitment was feasible because the required sample size was reached (sample size: 33 subjects; feasibility threshold: 30 subjects) with an adequate rate of recruited participants per site per month (recruitment rate: 8.25; feasibility threshold: >7.5). The retention and adherence of the trial were feasible, showing no dropouts from the trial (retention rate: 100%; feasibility threshold: >80%) and no non-compliance with the interventions (adherence rate: 100%; feasibility threshold: >80%). However, the adherence and retention rates must be interpreted with caution as only one treatment session was implemented. Future studies should evaluate if these feasibility rates remain acceptable in studies with more treatment sessions. Lastly, the study was feasible in terms of safety as no AEs were reported (adverse events rate: 0%; feasibility threshold: <6%). Therefore, no information could be provided on the type, severity, frequency, duration, and attributed cause of AEs as well as actions taken against them. In sum, the study protocol appears feasible, pending some modifications. The proposed changes to the study protocol are discussed subsequently.

The secondary objective was to evaluate the effect of HFP on HRV in a population of stressed subjects with MP compared to SHAM. The within-group changes from pre- to post-intervention showed a significant increase in RMSSD (*M* = 23.8; *SD* = 44.4; *p* = 0.036; Cohen’s *d* = −0.519), SDNN (*M* = 17.8; *SD* = 25.4; *p* = 0.009; Cohen’s *d* = −0.772), and LF/HF (*M* = 3.3; *SD* = 5.7; *p* = 0.026; Cohen’s *d* = −0.635) after HFP but not SHAM (*p* > 0.05). Further, there were significant within-group differences in HR from pre- to post-intervention, showing a reduction after both HFP (*M* = −2.8; *SD* = 2.2; *p* < 0.001; Cohen’s *d* = 0.303) and SHAM (*M* = −2.5; *SD* = 3.2; *p* = 0.009; Cohen’s *d* = 0.191). In contrast, there were no significant differences from pre- to post-intervention with regard to the SI and SD2/SD1 ratio in both the HFP and SHAM groups (*p* > 0.05). Hence, an autonomic effect of HFP can be assumed. Specifically, the increase in RMSSD and SDNN as well as the decrease in HR after HFP suggests an increase in PNS activity. In turn, no effect was reported for SI and SD2/SD1. Contrary to our hypothesis, LF/HF increased after HFP, which suggests an increase in SNS activity. However, the LF/HF ratio was criticised for not reflecting the sympatho-vagal balance [86]. Instead, it was argued that changes in the LF/HF ratio reflect the modulation of cardiac autonomic outflow by baroreflexes [87]. Taken together, this pilot study suggests that HFP produces a parasympathetic effect (which is evidenced by the HRV parameters RMSSD, SDNN, and HR but contradicted by the HRV parameters LF/HF, SI, and SD2/SD1) [10,36,70]. Subsequently, the findings are discussed and interpreted in the context of the current body of literature.

The tertiary objective was to calculate the sample size required for a future RCT with an Excel tool [81] based on Lachin (1981) [82]. The power analysis was based on the HRV parameter RMSSD and used a significance level of *p* < 0.05, statistical power of 80%, and a mean difference of 23.8 for HFP and 3.5 for SHAM to calculate a total sample size of 66 participants per group. Considering an estimated drop-out of 10%, a total of 72 participants should be recruited for a future RCT.

### 4.2. Comparable Literature

The current evidence regarding the effect of OMT on HRV in healthy subjects demonstrated that (1) HR decreased significantly [31] or did not change significantly [41]; (2) LF decreased significantly [40,44] or did not change significantly [43,50]; (3) HF increased significantly [28,31,41,43,44] or did not change significantly [50]; (4) LF/HF decreased significantly [31,40,43,44], increased significantly [40], or did not change significantly [47]; (5) SDNN increased significantly [43]; and (6) RMSSD increased significantly [41] or did not change significantly [47]. Far less is known about symptomatic subjects [36], and only a few studies have investigated the effect of OMT on HRV in patients with MP. More specifically, it was shown that OMT (compared to sham treatment) increases HF and RMSSD in patients with acute LBP [88], and it increases HF and RMSSD and decreases LF, LF/HF, and HR in patients with CLBP [42].

These findings (reported in the literature) are largely consistent with the results of the present pilot study, which demonstrated significantly decreased HR and significantly increased SDNN and RMSSD. In contrast, our pilot study reports a significant increase in LF/HF (reflecting an increase in SNS activity), whereas most previous studies demonstrated a significant decrease in LF/HF [31,40,43,44]. No information could be retrieved from the literature regarding the effect of OMT on SI and SD2/SD1. Therefore, this pilot study may provide preliminary evidence that OMT does not significantly change SI and SD2/SD1. Further, we did not collect data on LF and HF, which were previously shown to decrease (LF) [40,44] and increase (HF) after OMT [28,31,41,43,44]. Nonetheless, there are (methodological) limitations to acknowledge, which reduce the interpretability and generalisability of these findings.

### 4.3. Methodological Limitations

The methods used in this trial are underlined by limitations that need to be considered when interpreting the results. Firstly, some of the eligibility criteria used to recruit participants should be revised. Specifically, participants needed to be symptomatic and stressed to be eligible for trial participation. However, these criteria were not defined precisely enough. On the one hand, subjects needed to report musculoskeletal symptoms. However, the sample demonstrated heterogenous symptoms, showing MP with varying locations (e.g., shoulder or back pain) and durations (e.g., acute or chronic). Accordingly, the generalisability of these findings is minimised. In the future, a sample of subjects with a specific MP symptom (for example, CLBP) should be recruited. On the other hand, participants needed to show relevant levels of stress. However, there are no cut-off scores available for the PSS-10 [69]. Consequently, we used norm values from a German sample (*M* = 12.57, *SD* = 6.42) [69] to define the score required for a stressed population (PSS-10: ≥12). However, normative values from other countries ranged from 12 to 18 (China (*M* = 15.4, *SD* = 4.7), Sweden (*M* = 13.96, *SD* = 6.34), Mexico (*M* = 14.52–17.73), and the United States (*M* = 12.07–18.64)) [69,89,90,91,92]. Hence, it is questionable if our population can be defined as stressed. Nonetheless, the German norm values (*M* = 12.57, *SD* = 6.42) were markedly lower than the baseline stress levels reported in our study for both the HFP (*M* = 19.3, *SD* = 4.1) and SHAM groups (*M* = 18.4, *SD* = 4.7).

Secondly, the measures and outcomes were associated with limitations that need to be considered. The feasibility outcomes were limited by subjectivity (as the researchers rated the practicability), whereas the HRV outcomes were limited by various confounding factors. While some of these confounders were recorded in the present study (e.g., age, sex, BMI, and stress level), others were not (e.g., physical activity, respiratory rate, and consumption of meals, caffeine, nicotine, alcohol, or drugs) [36]. Regardless of whether these confounders were recorded or not, the effect on the results were not assessed (e.g., using subgroup analysis).

Thirdly, there are limitations to consider regarding the interventions. Sham controls are generally difficult to implement in manual therapy trials. Specifically, blinding is frequently compromised because the actual and sham treatments are not indistinguishable [93,94,95,96]. In this pilot study, there were both similarities and dissimilarities between the interventions. On the one hand, the interventions were similar regarding the characteristics of the practitioners (two women with similar age, experience, and training), context (treatment by an osteopath applied in-person to a passive recipient in the same treatment environment), and technique (time of contact (15–20 min), participant positioning (supine position), and type of touch (static) and pressure (light)). On the other hand, no information was recorded regarding the expectations and naivety of participants regarding the interventions and some characteristics of the technique were dissimilar with respect to the area of contact (touch was applied to the chest and back in the SHAM group and to the chest and back but also head and abdomen in the HFP group) and type of movement (the therapist initiated no movement in the SHAM group but followed micro-movement of the peripheral tissues in the HFP group). Furthermore, there are notable differences between the osteopathic and sham interventions, which may reduce the comparability between groups. HFP involves both manual approaches and modalities from MBIs [62], whereas the SHAM merely mimicked the manual approaches (e.g., touch) but did not involve or imitate MBI modalities (e.g., mindfulness and breathing). For example, subjects in the HFP group were instructed to observe and communicate bodily sensations and alter the breathing rate during treatment (which is integral to HFP), whereas verbal communication and breath control were not facilitated in the SHAM group. Consequently, these differences between the interventions may, on their own, explain some of the reported HRV improvements after HFP but not SHAM. Further, the amplified verbal communication between therapist and participant in the HFP group (compared to the SHAM group) might have improved the therapeutic alliance [97], which could (theoretically) have influenced the HRV findings [98]. Also, both groups were treated by different therapists, which may have swayed the results due to professional and inter-individual variability. Furthermore, the blinding concealment was not assessed and putative breaches in blinding could thus not be detected. Another shortfall is that the osteopathic technique used in this study (HFP) was newly developed by one of the authors (TL) and does not represent an established osteopathic technique nor osteopathic care as a whole.

Lastly, the study design and procedure show limitations that must be acknowledged. Pilot studies have implicit limitations like small sample sizes, which diminish the generalisability of findings. However, the aim of a pilot study is not to test the effectiveness of an intervention but rather to evaluate the feasibility of the study protocol [99]. Hence, generalisability refers to whether the study can be scaled-up for a future RCT (to avoid wasting resources) [100]. Further, this pilot study evaluated the therapeutic effect after only one treatment session, which does not represent clinical practice. For example, patients consult osteopaths for an average of two sessions in Switzerland [101]. In future studies, the number of treatment sessions should be increased. Another critique may be that the simple randomisation (drawing lots at each site) led to unbalanced groups (HFP: *n* = 18; SHAM: *n* = 15). In the future, block randomisation should be used to ensure equal group sizes, e.g., using a computer-generated allocation schedule (http://www.randomization.com, accessed on 3 January 2023). Further, the pilot study was merely single-blinded because double-blinding is not possible in manual therapy trials. Still, in future studies, the intervention providers and outcome assessors should be separated, and the outcome assessors should be blinded. In detail, manual therapists must necessarily be aware of the intervention they apply with their hands [84]. Lastly, the statistical methods may be extended to consider between-group differences at the post-intervention stage in future studies.

### 4.4. Future Directions

Despite increasing research interest and publications, various questions regarding the effect of osteopathic treatment on autonomic activity and the mechanisms underlying these changes remain open for future studies. On the one hand, the neurobiological mechanisms underpinning the (parasympathetic) effect of OMT on HRV are still under investigation [36]. It was hypothesised that OMT activates afferent C-tactile fibres (CTs), which leads to changes in interoceptive, inflammatory, and autonomic processes (e.g., increased interoceptive accuracy, decreased pro-inflammatory cytokines, and decreased sympathetic activity) [28,39,41,42,102,103]. More directly, osteopathic touch and manipulation may activate CTs that project to autonomic supraspinal nuclei involved with the control of cardiac ANS activity [36,39,41]. However, future research is needed to further understand the mechanisms leading to changes in HRV after OMT [39]. Similarly, the mechanisms of action through which MBI influences autonomic activity are not fully understood. A pilot study suggested that changes in breathing and muscle contraction may underlie the (parasympathetic) effect of MBI on HRV during stress; probably through enhanced interoception [104]. However, future research into the mechanisms of action underlying the effect of MBI on HRV is warranted. In sum, HFP may influence HRV through bottom-up mechanisms (e.g., OMT approaches involving touch to activate CTs) and top-down mechanisms (e.g., MBI approaches produce breathing and muscle contraction changes), which are arguably mediated through interoception. Still, further research on the mechanisms of action underlying OMT, MBI, and HFP is required.

On the other hand, the literature on the autonomic effect of manual treatments is largely limited to the short-term measurements and reports inconsistent findings regarding the direction of the autonomic effect (sympathetic versus parasympathetic), depending on the type of manual technique used (e.g., manipulation versus myofascial release) and the kind of body region treated (e.g., thoracic versus lumbar spine) [36,37]. Future research should use multiple autonomic measures (e.g., HRV, thermography, and skin conductance) and correlate them with clinical outcomes (e.g., pain and function) [36]. To reiterate, patients with MP show altered HRV, which may be reversed by OMT. Thus, it was hypothesised that changes in HRV may mediate the effect of OMT on MP [105]. Through this lens, changes in autonomic activity may be a mechanism of action underlying OMT. In the future, studies may assess if HRV can be used as a biomarker to predict changes in MP after OMT (e.g., using mediation analysis). Hence, future studies on HFP and HRV should consider the following methodological changes to the population and outcomes.

First, we propose that future studies should investigate the effect of HFP on HRV in patients with different MP symptoms. A meta-analysis found that patients with chronic pain conditions show significantly reduced HRV (HF ↓, SDNN ↓, LF/HF ↑, and no difference in LF and RMSSD) compared to healthy controls (HCs), suggesting decreased parasympathetic activity and/or increased sympathetic activity [106]. For example, reduced HRV (compared to HCs) was reported in patients with LBP (LF ↑, HF ↓) [12], neck pain (NP) (LF ↓, SDNN ↓), headache disorders (HF ↓, RMSSD ↓) [107], irritable bowel syndrome and inflammatory bowel disease (HF ↓, RMSSD ↓) [108], and fibromyalgia (HF ↓, SDNN ↓, LF ↑, LF/HF ↑, and no difference in LF and RMSSD) [109]. In turn, OMT was shown to improve pain and disability in patients with LBP [25] and NP [110] and seems to improve fibromyalgia [111,112,113,114,115], irritable bowel syndrome [116], and inflammatory bowel disease [117] as well as pain frequency, intensity, duration, and disability in headache disorders [118,119]. Hence, it is recommended to investigate the effect of HFP on HRV in these conditions.

Second, we suggest extending the outcome measures in future studies on the effect of HFP on HRV. This should include clinical outcome measures to assess pain and disability in patients with MP, using the appropriate tools depending on the symptoms (e.g., numeric rating scale and Oswestry disability index in patients with LBP) [120,121]. Thereby, the effect of HFP on the symptoms can be assessed secondarily. Further, we speculate that stress levels may be reduced by HFP. Indeed, stress reductions have been reported after osteopathic [30] and mind–body interventions [122]. The effect of HFP on stress levels could be evaluated in future studies using self-report (e.g., PSS-10) and biomarkers (e.g., cortisol levels). Furthermore, it is hypothesised that HFP may improve the patients quality of life due to the positive effects that have been reported after osteopathic treatment [123] and mind–body interventions [124]. It is proposed to include the SF-36 as a secondary measure for quality of life in future studies. Lastly, it is unclear how HFP may produce the effect on HRV. Arguably, the interoceptive system may be involved. A possible mechanism would be the activation of CT fibres that project to autonomic supraspinal nuclei, which are involved in the control of cardiac autonomic activity [36,39,41]. Thus, it may be beneficial to include outcome measures to investigate the effect of HFP on interoceptive function (e.g., interoceptive accuracy and sensibility) by means of performing a heartbeat tracking task [125] and filling in the second version of the multidimensional assessment of interoceptive awareness questionnaire [126].

## 5. Conclusions

Taken together, this pilot study can be considered feasible as the recruitment, retention, adherence, and adverse events rates were acceptable. As predicted, HFP (not SHAM) was shown to significantly increase SDNN and RMSSD in stressed subjects with MP, which suggests a parasympathetic effect. In contrast, HR significantly decreased after both HFP and SHAM, SI and SD2/SD1 did not change significantly after both HFP and SHAM, and LF/HF increased significantly after HFP but not SHAM, which contradicts the predicted parasympathetic effect. A future RCT is needed to validate or falsify these results, which should modify the methods of the study protocol according to the insights gained from this pilot study and recruit a total sample of 72 participants (*n* = 36) as determined by the power analysis (Appendix C).

## Figures and Tables

**Table 2:** Baseline data.

Parameter	HFP (*n* = 18) ^a^	SHAM (*n* = 15) ^a^	Significance ^b^
Age (years)	49.7 ± 12.6	45.3 ± 14.5	0.673
Sex (m/f ratio)	6:12	7:8	0.493 ^†^
Height (cm)	172.5 ± 8.2	175.0 ± 8.5	0.929
Weight (kg)	67.9 ± 14.2	74.2 ± 11.6	0.578
Stress (PSS-10 score)	19.3 ± 4.1	18.4 ± 4.7	0.796
HR (bpm)	64.7 ± 10.3	66.4 ± 11.1	0.598
RMSSD (ms)	43.0 ± 19.9	58.1 ± 38.9	0.007 **
SDNN (ms)	48.4 ± 17.7	57.4 ± 28.7	0.036 *
SI (pts)	185.5 ± 119.4	183.9 ± 120.6	0.933
LF/HF (ratio)	2.6 ± 3.5	1.6 ± 1.2	0.795
SD2/SD1 (ratio)	−1.1 ± 1.0	−1.3 ± 1.1	0.173

Legend: ^a^ = Data are reported as mean ± standard deviation; ^b^ = Data are reported as *p*-values (from independent two-sample Student’s *t*-test or Chi-square test; the latter was marked with ^†^); * = *p* < 0.05; ** = *p* < 0.01. Abbreviations: m/f ratio = male-to-female ratio; cm = centimetre; kg = kilogram; PSS-10 = perceived stress scale, 10-item version; HFP = heart-focused palpation (intervention group); SHAM = sham treatment imitating HFP (control group).

**Table 3:** Feasibility rates (primary outcomes).

Outcome	Calculation	Rate
Recruitment rate	33 [subjects] ÷ 2 [sites] ÷ 2 [months]	8.25 ^a^
Retention rate	33 [subjects analysed] ÷ 33 [subjects recruited] × 100	100 ^b^
Adherence rate	33 [sessions attended] ÷ 33 [sessions available] × 100	100 ^b^
Adverse events rate	0 [adverse events] ÷ 33 [subjects] × 100	0 ^b^

Legend: ^a^ = Data are reported as numbers (N [subjects per site per month]); ^b^ = Data are reported as percentages (%).

**Table 4:** Heart rate variability (secondary outcomes).

Parameter	Group	Pre-Intervention ^a^	Post-Intervention ^a^	Difference ^a^	Difference ^b^	Significance ^c^	Effect Size ^d^
HR (bpm)	HFP	64.7 ± 10.3	61.9 ± 10.0	−2.8 ± 2.2	4.3	<0.001 **	0.303
	SHAM	66.4 ± 11.1	63.5 ± 9.0	−2.5 ± 3.2	4.4	0.009 *	0.191
RMSSD (ms)	HFP	43.0 ± 19.9	66.8 ± 53.6	23.8 ± 44.4	55.4	0.036 *^,†^	−0.519
	SHAM	58.1 ± 38.9	61.6 ± 33.9	3.5 ± 20.6	6.0	0.521 ^†^	−0.094
SDNN (ms)	HFP	48.4 ± 17.7	66.2 ± 29.0	17.8 ± 25.4	36.8	0.009 *	−0.772
	SHAM	57.4 ± 28.7	63.7 ± 25.7	6.3 ± 18.2	11.0	0.200	−0.229
SI (pts)	HFP	185.5 ± 119.4	141.1 ± 132.4	−44.4 ± 104.4	23.9	0.089	0.384
	SHAM	183.9 ± 120.6	148.5 ± 79.9	−35.4 ± 69.6	19.3	0.069	0.292
LF/HF (ratio)	HFP	2.6 ± 3.5	5.8 ± 6.5	3.3 ± 5.7	123.1	0.026 *^,†^	−0.635
	SHAM	1.6 ± 1.2	2.0 ± 1.3	0.4 ± 1.6	25.0	0.359 ^†^	−0.306
SD2/SD1 (ratio)	HFP	−1.1 ± 1.0	−1.2 ± 1.6	−0.1 ± 1.0	9.1	0.527	0.103
	SHAM	−1.3 ± 1.1	−1.2 ± 1.1	0.0 ± 0.74	7.7	0.875	−0.160

Legend: ^a^ = Data are reported as mean ± standard deviation; ^b^ = Data are reported as percentages; ^c^ = Data are reported as *p*-values (from paired Student’s *t*-test or Wilcoxon signed-rank test; the latter was marked with ^†^); ^d^ = Data are reported as Cohen’s *d*; * = *p* < 0.05; ** = *p* < 0.01.

## Data Availability

The data presented in this study are available on request from the corresponding author.

## Supplementary Materials

The following supporting information can be downloaded at https://www.mdpi.com/article/10.3390/healthcare12020138/s1, Table S1: CONSORT checklist.

## Appendix A. Perceived Stress Scale—10 Items

The PSS-10 is a self-report questionnaire with ten questions rated on a 5-point Likert scale [128]. The total score ranges from 0 (no stress) to 40 (high stress) [69]. The German version of the PSS-10 was used, which is a valid and reliable tool to measure stress [69,128]. The PSS-10 does not have a diagnostic cut-off value [69]. However, the norm value in Germany is ~12 (*M* = 12.57, *SD* = 6.42) [69]. Consequently, we defined a stressed population as showing a PSS-10 score of ≥12.

## Appendix B. Heart-Focused Palpation

Notably, it is not suggested that osteopaths can palpate the heart in a literal sense. While some osteopaths believe that the heart can be palpated manually [129], others have opposed these unsubstantiated claims [130]. Importantly, HFP is not a diagnostic test but a therapeutic technique. Consequently, we consider the “palpation of the heart region” not as a diagnostic test to identify anatomical structures, but rather as a therapeutic technique that uses modalities from OMT and MBIs to ostensibly improve self-perception, relaxation, and autonomic regulation.

## Appendix C. Highlights

- The study was feasible regarding the recruitment, retention, adherence, and safety.
- A power analysis revealed that 72 participants are needed for a future RCT.
- HFP improved HRV in stressed subjects with musculoskeletal pain.
- LF/HF, SDNN, and RMSSD increased, SI and SD2/SD1 did not change, and HR decreased.
- The results suggest a parasympathetic effect of HFP.
